# Ambulatory Medical Assistance - After Cancer (AMA-AC): A model for an early trajectory survivorship survey of lymphoma patients treated with anthracycline-based chemotherapy

**DOI:** 10.1186/s12885-015-1815-7

**Published:** 2015-10-24

**Authors:** Gisèle Compaci, Manuela Rueter, Sébastien Lamy, Lucie Oberic, Christian Recher, Maryse Lapeyre-Mestre, Guy Laurent, Fabien Despas

**Affiliations:** 1 Department of Hematology - Internal Medicine, Toulouse University Hospital, Cancer University Institute of Toulouse Oncopole, Toulouse, France; 2 INSERM Unit 1027 (The French National Institute of Health and Medical Research), Faculty of Medicine, Toulouse, France; 3 Service of Medical and Clinical Pharmacology, Center of Pharmacovigilance, Pharmaco-epidemiology and Information on Drugs, Toulouse University Hospital, 37 Allées Jules Guesde, 31000 Toulouse, France; 4 Department of Epidemiology, Health Economics and Public Health, Faculty of Medicine, University of Toulouse III Paul Sabatier, Toulouse, France; 5 INSERM Unit 1037 (The French National Institute of Health and Medical Research), Center of Cancer Research, Toulouse, France; 6 Laboratory of Medical and Clinical Pharmacology Faculty of Medicine, University III Paul Sabatier, Toulouse, France

**Keywords:** Cancer survivorship, Lymphoma, Anthracycline-based chemotherapy, Shared care model

## Abstract

**Background:**

Cancer survivorship has emerged as an important aspect of oncology due to the possibility of physical and psychosocial complications. The purpose of this study was to assess the feasibility of the Ambulatory Medical Assistance for After Cancer (AMA-AC) procedure for monitoring lymphoma survivorship during the first year after chemotherapy.

**Methods:**

AMA-AC is based on systematic general practitioner (GP) consultations and telephone interventions conducted by a nurse coordinator (NC) affiliated to the oncology unit, while an oncologist acts only on demand. Patients are regularly monitored for physical, psychological and social events, as well as their health-related quality of life (HRQoL). Inclusion criteria were patients newly diagnosed with non-Hodgkin or Hodgkin lymphomas, who had been treated with anthracycline-based chemotherapy and were in complete remission after treatment.

**Results:**

All 115 patients and 113 collaborating GPs agreed to participate in the study. For patients who achieved one year of disease-free survival (*n* = 104) their assessments (438 in total) were fully completed. Eleven were excluded from analysis (9 relapses and 2 deaths). The most frequent complications when taking into account all grades were arthralgia (64.3 %) and infections (41.7 %). About one third of patients developed new diseases with cardiovascular complications as the most common. Psychological disorders such as anxiety, depression and post-traumatic stress disorder were diagnosed in 42.6 % of patients. The data collected showed that Hodgkin lymphoma patients, females, and patients with lower HRQoL (mental component) at study entry were at greater risk for developing at least one psychological disorder.

**Conclusion:**

This study showed that AMA-AC is a feasible and efficient procedure for monitoring lymphoma survivorship in terms of GP and patient participation rates and adherence, and provides a high quality of operable data. Hence, the AMA-AC procedure may be transferable into clinical daily practice as an alternative to standard oncologist-based follow-up.

**Electronic supplementary material:**

The online version of this article (doi:10.1186/s12885-015-1815-7) contains supplementary material, which is available to authorized users.

## Background

Cancer survivorship has recently emerged as an important aspect of cancer patient trajectory. Cross-sectional studies and registry-based data analyses have documented that cancer survivors present with a variety of troubles that can lead to a decrease in their health-related quality of life (HRQoL). Compared to that of solid tumors (notably breast cancers), lymphoma survivorship has received little attention, but studies examining the course of morbidity in Non-Hodgkin lymphoma (NHL) and Hodgkin lymphoma (HL) survivorship have revealed that these patients experience psychological disorders (e.g., anxiety, depression, post-traumatic stress disorder [PTSD]) [1–3], delayed return to work [4], and a subsequent decrease in their HRQoL [3, 5]. Beside these complications, other severe concerns include the development of cardiovascular diseases and second malignancies, while relapse also remains possible, especially during the first 24-months post-therapy [6].

Since the development of therapies to treat NHL and HL patients, the number of survivors has increased and is now estimated at 170,000 cases in the USA [1], 38,000 in Germany [7] and 35,000 in France [8]. However, one of the main difficulties in managing cancer survivorship is how to detect complications such as those listed above. Addressing this requires a consideration of the role of each care provider who is in contact with cancer survivorship patients. In theory, cancer patient survivorship surveillance involves a fair and effective collaboration between oncologists, general practitioners (GPs) and potentially other specialists depending on the nature of any complications. Oncologist contact is mainly through scheduled regular visits whereas GPs mainly operate as the first point of contact for patients experiencing symptoms related or not to cancer or treatment. This so-called “shared care” model has been supported by public health decision-makers and is largely favored by GPs. However, this model has been seriously questioned on the basis of several considerations related to both GPs and hospital insufficiencies. When surveyed, GPs reported not feeling comfortable with cancer survivorship management [9]. In general, GPs are thought to be poorly informed about the nature and risk of late complications, especially delayed adverse effects of therapies [10, 11], and they are not familiar with the psychological and social aspects of cancer patients [9]. Thus, it is not surprising that the majority of patients prefer to be followed-up by their oncologist rather than their GP, as has been reported for breast cancer survivors [12]. These considerations may also explain why the shared care model is less popular in the oncologist community [13]. However, it has become more and more evident that oncologist-based survivorship follow-up also suffers from a number of flaws since, despite being the most common model used, it appears that hospital follow-up is cursory and poorly adapted to the detection and graduation of psychological disorders, professional difficulties and HRQoL degradation [14]. Moreover, relapse or associated diseases, if they occur, are often diagnosed outside of a review visit [15]. Thus, the standard hospital-based protocol of appointments is possibly not the most productive and effective health care model for cancer survivorship. In a large recent survey dealing with gynecological cancer follow-up in the United Kingdom, Leeson et al. described a switching of practices, with traditional follow-up being replaced by telephone follow-up in 25 % of cases [16].

Telephone intervention, generally performed by specialized nurses (nurse coordinators [NC]), has been used at different stages in the cancer patient trajectory, including the early steps of diagnosis (the concept of a “Patient Navigator”) [17], during the management of advanced cancers [18], and whilst undergoing psychotherapy treatment for PTSD [19]. Most of these studies have shown clinical benefits. In a previous report, we described the Ambulatory Medical Assistance (AMA) project, a new modality of patient management for diffuse large B-cell lymphoma (DLBCL) patients undergoing therapy with R-CHOP or R-CHOP-derived protocols. AMA is based on scheduled appointments for patient phone calls from home with a NC during their active treatment phase. AMA has been found to be feasible and very effective in both its triage function and in saving medical time [20]. Moreover, it appears that AMA not only generates great satisfaction among patients and caregivers but has also improved chemotherapy observance, reduced secondary hospitalization and, perhaps, decreased the toxic death rate [20].

Based on the success of AMA, we designed the AMA-AC (Ambulatory Medical Assistance - After Cancer) model, a variant of the shared care model which is based on close collaborations between a NC and the patient’s GP for the surveillance of lymphoma survivors. The present study is based on an ongoing prospective cohort of 115 lymphoma patients treated with anthracycline-containing regimens. This study was aimed at investigating whether AMA-AC is a feasible procedure for monitoring a patient’s physical, psychological and social events during the first year after therapy.

## Methods

### AMA-AC program recruitment

To be selected for the AMA-AC program, volunteers must have received treatment for B- or T-cell derived NHL or advanced HL, with their first-line of treatment consisting of an anthracycline-based therapy (i.e., CHOP21, R-CHOP21, R-CHOP-derived, ABVD or BEACOPP) at the Toulouse University Hospital. They also must have achieved a complete response according to the Cheson’s criteria [21], and been followed-up by a GP who had agreed to participate in the program. Patients under 18 years of age at diagnosis, or who were physically and/or mentally unable to participate in the program were not included. The study has been approved by the ethical committee of the Toulouse University Hospital and all participants gave their written informed consent. Between 1^st^ November 2011 and 1^st^ November 2013, 115 patients joined the AMA-AC program.

### AMA-AC program design

**Fig. 1:**
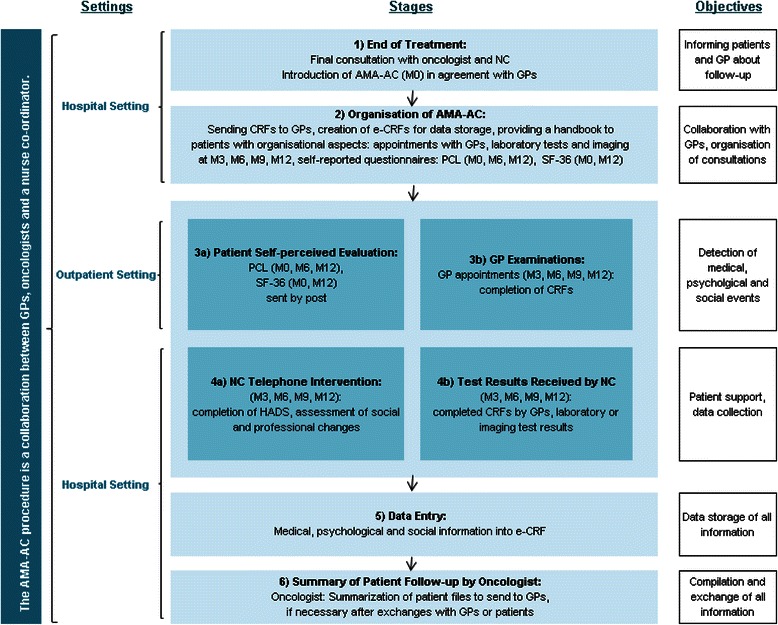
Scheme of the AMA-AC procedure

The program is presented in detail in Fig. 1. Briefly, the AMA-AC program consisted of one initial visit to an oncologist in the presence of a NC. The patient received a handbook which contained all information related to the AMA-AC procedure and a calendar for the scheduled regular appointments with their GP (physical visit) and with the NC (phone call at patient’s home). This handbook was also forwarded by e-mail to the patient’s GP who in addition received a clinical report form (CRF) specially prepared to help detect any physical events. The AMA-AC program consisted of quarterly follow-up assessments for monitoring any medical, psychological and social events. It encompassed GP appointments, self-perceived evaluation of HRQoL and mental health, and phone calls conducted by the NC. The CRF contained 41 items related to three groups of symptoms: symptoms compatible with a relapse, symptoms suggesting previously undocumented comorbidities (e.g., cardiovascular complications), and symptoms classified as adverse drug effects (e.g., neuropathy). Importantly, the informed consent form clearly stated that the program did not include any systematic appointments with an oncologist; however the patients were able to consult their oncologist on demand at any time at the hospital. Throughout the program the CRF was completed by the GP during each GP consultation and forwarded by e-mail to the NC. Regular biological analyses (i.e., blood cell count, liver and kidney function, C-reactive protein, lactate dehydrogenase, protein electrophoresis) were also performed at a location near to the patient’s home and forwarded to the NC. Information about psychological events was gathered through patient self-evaluation of health outcome and through NC phone calls. In addition, during the telephone interview the NC questioned patients regarding their social and professional status or any other changes (e.g., return to work, disability pensioning, personal resources). The resulting file, compiled by the NC, included physical, psychological, social and professional sections. The NC was in charge of forwarding this data to the oncologist, who summarized all the information and if necessary would call the patient or their GP for clarification, or as a last degree would call the patient in for a visit at the hospital. In each case, the oncologist then forwarded his conclusion to the GP by post. In some cases, symptom detection required referrals to additional clinical and psychosocial providers. For the most part these specialists were designated by the GP and worked in private practice. The NC (or oncologist) was responsible for making contact with these specialists, planning appointments, and addressing all relevant information.

### Data collected by the AMA-AC program

#### Initial patient characteristics

Individual, disease-related and treatment-related initial characteristics were collected. Individual characteristics included gender, age at inclusion into the AMA-AC program (M0 = Month 0), health insurance coverage, familial status (i.e., whether patients lived alone or not), level of education, occupational status, and salary per month. Disease-related characteristics included histology type, Ann Arbor stage, Eastern Cooperative Oncology Group (ECOG) performance status, Charlson comorbidity index (CCI) [22, 23], prognostic index with regard to histological type: the revised international prognostic index (IPI) for DLBCL [24], the follicular lymphoma prognostic index (FLIPI) for follicular lymphoma [25], and the Hasenclever international prognostic index for advanced HL [26]. Treatment characteristics corresponded to the first-line chemotherapy regimens dichotomized as “conventional” for CHOP21, R-CHOP21, ABVD, and R-mini CHVP and “intensified” for R-ACVBP, irrespective of whether this was followed or not by autologous hematopoietic stem cell transplantation (ASCT), R-COPADM and BEACOPP.

### Medical events

Physical events were assessed in the 41-item CRF completed during GP appointments and included symptoms potentially related to relapse, newly diagnosed comorbidities, and adverse drug effects (see Additional file 1 for the complete CRF).

Psychological disorders included anxiety, depression and post-traumatic stress disorder (PTSD). Anxiety and depression were assessed by quarterly phone calls (M3, M6, M9 and M12) according to the French version of the 14-item Hospital Anxiety and Depression Scale (HADS) [27, 28], which is divided into two subscales: anxiety (HAD-A) and depression (HAD-D). A score between 0 and 21 was calculated for each subscale with a higher score indicating a higher level of anxiety or depression. For each quarter, the overall incidence of anxiety and depression was calculated as the ratio of new cases (defined by a HAD-D or HAD-A score above 8) over the number of patients at risk at the beginning of the study period (i.e., those free of anxiety or depression). The prevalence of anxiety and depression at each quarter was also computed as the ratio of total number of cases (defined by a HAD-D or HAD-A score above 8) over the total number of patients followed in the period. However, although the self-perceived questionnaire measured the extent of anxiety or depressive symptoms experienced, this could not replace clinical diagnosis, therefore GPs were contacted in cases of noticeable values and, if needed, patients were referred to specialists. PTSD was measured using the French version of the PTSD checklist (PCL) [29–31], mailed to the patients’ homes for assessment at M6 and M12. The PCL assessed the presence of PTSD symptoms by scoring responses related to three symptom groups: re-experiencing, avoidance and hyper-arousal. The PCL is a 17-item self-reporting checklist measuring PTSD. It is delineated in the fourth edition of the Diagnostic and Statistical Manual of Mental Disorders (DSM-IV) [32], and was adapted for the diagnosis and treatment of cancer. Patients were asked to rate their experience of each of the 17 symptoms on a five point scale, from 1 (not at all) to 5 (extremely) during the previous month. The PCL total scores ranged from 17 to 85. Patients with a total score ≥44 were considered to have PTSD. In addition, a computer tomography (CT) scan was performed on all patients at M6.

### Complementary information

Professional and social parameters were also gathered during the quarterly phone interviews, including any return to work, changes in home address and changes in cohabiting status.

HRQoL was assessed using the self-reported French version of the SF-36 [33–36], mailed to the patients’ homes, at M0 and M12. The 36 items on this list were distributed into two subscales: the Physical Component Score (PCS) and the Mental Component Score (MCS), scored from 0 (poor) to 100 (excellent).

### Data collection and analysis

An anonymized database was used to collect all information related to the AMA-AC program. This database was secured and managed by an external service device in accordance with *ad hoc* regulatory committees. In order to determine the strength of the relationship between each of the variables (PTSD, HAD-Depression, HAD-Anxiety, SF36-MCS, and SF36-PCS scores) measured at M0, M3, M6, M9 and M12, we generated a Pearson correlation matrix. A correlation coefficient of 1.0 indicated a positive correlation and a value of −1.0 indicated a negative correlation. According to the guidelines by Cohen et al. [37], a correlation coefficient between 0.10 and 0.29 corresponds to a small strength of correlation, 0.30 to 0.40 denotes a medium correlation and 0.50 to 1.0 signifies a high correlation between the variables. We implemented a multivariate logistic regression model adjusted for variables statistically associated with the outcome in bivariate analyses with a risk alpha of 20 %, except for the first-line chemotherapy regimen which was forced in the model. Interactions between the covariates were verified for each model. Assumptions and model fit were measured using the Hosmer and Lemshow test. A two-sided p-value <0.05 was considered as statistically significant for the multivariate model. Statistical analyses were performed using SAS®software version 9.4 (SAS institute, Cary, NC).

## Results

### Implementation of AMA-AC

A total sample of 115 patients, followed by 113 GPs (2 of whom each monitored 2 patients), entered into the AMA-AC program. Patient characteristics are listed in Table 1, data were exhaustive for the characteristics assessed, with the exceptions of salary (77 % complete), Ann Arbor stage and prognosis index (97 % complete for both). Histology subtypes were as follows: diffuse large B-cell lymphoma (DLBCL): 64 patients (55.7 %), follicular lymphoma (FL): 27 patients (23.4 %), Hodgkin lymphoma: 18 patients (15.7 %), and other non-Hodgkin lymphoma (NHL): 6 patients (5.2 %). All GPs agreed to participate in the program but 11 patients were excluded due to relapse (*n* = 9) and death (*n* = 2) related to causes other than the primary cancer. Thus, a total of 104 patients were followed-up for at least one year. The AMA-AC procedure consisted of 438 patient assessments: 115 at M3, 113 at M6, 106 at M9, and 104 at M12. The auto-questionnaires (SF-36, PCL) were completed at home and sent to the NC in all cases. The GPs returned each CRF (100 % validity), and reported that these required about 15 min to complete. The median time for the nurse-led phone calls was 30 min. The oncologists spent a median time of 10 min for the synthesis and summary letter (via voice recognition dictation). Altogether, the procedure represented 55 min per quarter (i.e., 220 min per patient per year of follow-up). A significant gain of time was obtained through auto-evaluation of the PCL and SF-36 by the patient. According to the AMA-AC procedure, patients were able to visit an oncologist on demand. Among the 104 patients free of relapse and alive at M12, only 6 patients (6.5 %) returned to the hospital during the first 12 months of follow-up for the following reasons: fear of relapse based on imaging or subjective symptoms (which were not confirmed; *n* = 4) and delayed neutropenia (post-rituximab neutropenia) requiring bone marrow analysis (*n* = 2).

**Table 1:** Characteristics of the 115 patients included in the AMA-AC program

Patient characteristics at diagnosis/entry to AMA-AC	(*n* = 115)
Gender Men (n;%)	64 (55.7 %)
Age (years)	
Mean (Min; Max)	55.0 (22.0; 87.0)
Median	58
Health insurance (n;%)	
General health system	104 (90.4 %)
Others (Agriculture, freelancers)	11 (9.6 %)
Cohabiting status (n;%)	
Living together (married, living in partnership)	87 (75.6 %)
Living alone (single, divorced, widowed)	28 (24.4 %)
Level of education (n;%)	
Lower educational status (≤high school degree)	64 (55.7 %)
Higher educational status (>high school degree)	51 (44.4 %)
Occupational status (n;%)	
In activity (employed)	61 (53.0 %)
Without activity (without employment, retired, unemployed)	54 (47.0 %)
Salary net/month (n;%) (*n* = 86)	
No salary	3 (3.5 %)
<380€ - 1070€	20 (23.3 %)
>1070€ - 1830€	24 (27.9 %)
>1830€ - 2290€	12 (14.0 %)
>2290€ - 4570€	27 (31.4 %)
Disease-related characteristics	
Histology (n;%)	
Diffuse large B-cell lymphoma (DLBCL)	64 (55.7 %)
Other NHL	33 (28.6 %)
Hodgkin lymphoma	18 (15.7 %)
Ann Arbor stage (n;%) (*n* = 112)	
I/ II	25 (22.3 %)
III/ IV	87 (77.7 %)
Performance status (n;%)	
≤ 1	96 (83.5 %)
≥ 2	19 (16.5 %)
Charlson comorbidity index (n;%)	
0	88 (76.5 %)
1	9 (7.8 %)
≥ 2	18 (15.7 %)
Prognosis (according to IPI, FLIPI, IPS) (*n* = 112)	
Good	19 (16.52 %)
Medium	59 (51.30 %)
Bad	24 (20.87 %)
Treatment-related characteristics	
Type of treatment line (n;%)^a^	
Conventional	84 (73.0 %)
Intensified	31 (27.0 %)

*Abbreviations*: *IPI* International Prognostic Index; *FLIPI* Follicular Lymphoma International Prognostic Index; *IPS* International Prognostic Score (Hasenclever Index)

^**a**^Type of treatment line: Conventional: CHOP21: 4 (3.5 %), R-CHOP21: 65 (56.5 %), R-mini-CHOP: 3 (2.6 %), ABVD: 12 (10.4 %); Intensified: R-ACVBP: 24 (20.9 %), R-COPADM: 1 (0.9 %), BEACOPP: 6 (5.2 %)

### Physical events during follow-up

#### Treatment-related complications

The prevalence of physical disorders at each quarterly assessment are depicted in Table 2. For the entire one-year follow-up, the most frequent complications when taking into account all grades were: arthralgias (64.3 %) and infections (41.7 %), the latter being most often associated with mild hypogammaglobulinemia. Indeed, although 47.0 % of patients displayed immunoglobulin levels lower than 8 g/L, severe hypogammaglobulinemia (<3 g/L) was rare (2.6 %). A third of infections were pneumonia or sinusitis. Herpes zoster was infrequent (*n* = 3). Neuropathies due to vincristine or vinblastine were identified in 24.3 % patients, with all grades included in this, however these resolved over time (16.3 % at M12). As an unexpected finding, gastric symptoms were frequent (17.4 % of patients). Among the patients with gastric symptoms, endoscopy was performed in about one third. Libido changes (most often in males) were observed in 14.8 % of patients. Among men, erectile dysfunction was observed in 20/64 patients (31.3 % of patients. Forty percent of male patients with erectile dysfunction were treated with tadalafil. The occurrence of symptomatic osteoporosis during the first 12 months of survivorship was also common (13.3 % of patients; exclusively females). We found no influence of histology subtype (DLBCL, FL or HL), on the distribution of treatment-related complications, with the exception of hypogammaglobulinemia which was more frequently observed for DLBCL.

**Table 2:** Monitored treatment-related complications and comorbidities during one year of follow-up

	Phone call 1		Phone call 2		Phone call 3		Phone call 4			
Prevalence of complications	Month 3 (*n* = 115)		Month 6 (*n* = 113)		Month 9 (*n* = 106)		Month 12 (*n* = 104)		Total	(*n* = 115)
	n	%	n	%	n	%	n	%	n	%
Treatment-related complications										
Neuropathy										
Peripheral	26	22.6 %	24	21.2 %	22	20.7 %	17	16.3 %	28	24.3 %
Central	1	0.9 %	2	1.8 %	0	0.0 %	2	1.9 %	2	1.7 %
Infections										
Pulmonary	14	12.2 %	8	7.1 %	8	7.5 %	8	7.7 %	38	33.0 %
Ear, nose and throat	5	4.4 %	5	4.4 %	8	7.5 %	5	4.8 %	23	20.0 %
Urinary	4	3.5 %	4	3.5 %	3	2.8 %	2	1.9 %	13	11.3 %
Hypogammaglobulinaemia	15	13.0 %	36	31.9 %	43	40.6 %	31	29.8 %	54	47.0 %
Gastritis/ulcer	14	12.2 %	17	15.0 %	18	17.0 %	11	10.6 %	20	17.4 %
Arthralgia	52	45.2 %	57	50.4 %	47	44.3 %	44	42.3 %	74	64.3 %
Libido decrease	16	13.9 %	7	6.2 %	10	9.4 %	10	9.6 %	17	14.8 %
Erectile dysfunction (*n* = 64)	11	17.2 %	6	9.5 %	7	10.9 %	6	10.2 %	20	31.3 %
Osteoporosis	9	7.8 %	10	8.9 %	9	8.5 %	12	11.5 %	15	13.3 %
Comorbidities										
Cardiovascular complications (≥1/phone call)	6	5.2 %	4	3.5 %	6	5.7 %	10	9.6 %	16	13.9 %
Disorders of thyroid gland	5	4.4 %	5	4.4 %	4	3.8 %	7	6.7 %	7	6.1 %
Disorders of prostate (*n* = 64)	3	4.7 %	2	3.2 %	2	3.1 %	2	3.1 %	3	4.7 %
Second cancer	1	0.9 %	1	0.9 %	1	0.9 %	2	1.9 %	4	3.5 %

#### Relapse

Within the first year of survivorship nine patients relapsed: (*n* = 3 before M6, *n* = 6 between M6 and M12). In all cases the relapse was suspected by the patients themselves and was confirmed by clinical symptoms and examination by GPs. Consequently, patients were re-examined by an oncologist on demand to confirm the relapse by biopsy and histological analysis at the hospital. The CT scans performed at M6 (*n* = 108 examinations) played no part in detecting relapses (data not shown).

#### Newly-diagnosed comorbidities

About one third of patients developed new diseases during the early stages of survivorship (Table 2). The most frequent complications were cardiovascular diseases (*n* = 16) with sometimes more than one per patient: thromboembolic diseases (*n* = 5), arrhythmias (*n* = 9), atherosclerotic heart disease resulting in myocardial infarction (*n* = 1), severe pericarditis (*n* = 1) and arterial hypertension (*n* = 1). The thyroid was also affected in 6.1 % of patients: thyroid insufficiency (*n* = 3, detected by biological testing) and thyroid nodules (*n* = 4) among which one cancer was discovered. Prostatic adenomas or prostatitis were less common (4.7 % of patients). One patient who presented as a relapse in fact had a secondary lymphoma (marginal zone lymphoma complicating a follicular lymphoma). The CT scan performed at M6, although ineffective at detecting relapses, raised major concerns in 4 out of 111 patient examinations (3.6 %), and led to the diagnosis of one pancreatic cancer, one intraductal papillary mucinous neoplasm of the pancreas (preneoplasic lesions), one pulmonary embolism, and one asymptomatic choledocallithiasis. Overall, among 106 patients not showing a relapse, 11 of them (10.4 %) developed serious non-haematological diseases within the first year of follow-up, among which there were 3 adenocarcinomas.

### Non-physical events during follow-up

#### Psychological disorders (PTSD, anxiety or depression)

**Fig. 2:**
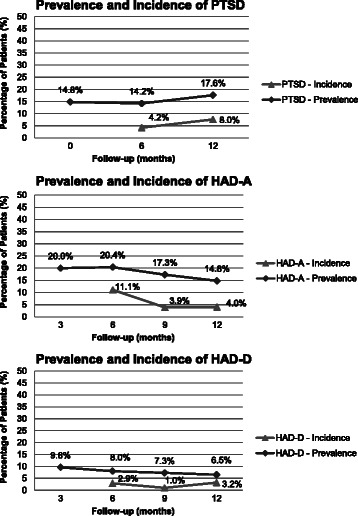
Prevalence and incidence of PTSD (*top*), measured every 6 months, and anxiety (*middle*) and depression (*bottom*) evaluated every 3 months

During the first phone call (M3) the prevalence of anxiety was as high as 20.0 % but decreased over time (14.8 % at M12). The prevalence of depression was less frequent (9.6 % at M3 and 6.5 % at M12). The prevalence of PTSD ranged between 14.8 % of 115 patients at M0 and 17.6 % of 104 patients at M12 (Fig. 2). Over the first 12 months, 42.6 % of patients presented with at least one of the three psychological disorders (anxiety, depression or PTSD): 20.8 % patients (*n* = 24/115) had one disorder, 12.2 % (*n* = 14/115) had two and 9.6 % (*n* = 11/115) had all three.

#### Health-related Quality of Life (HRQoL)

**Fig. 3:**
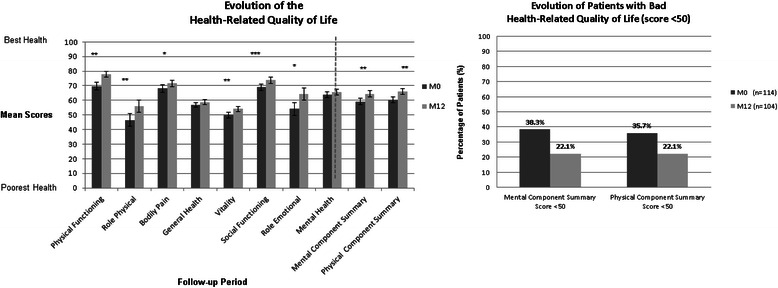
Health-Related quality of life (SF-36) evaluation with the SF-36 at the entry of AMA-AC (*n* = 114 patients) and after 12 months (*n* = 104 patients)

HRQoL was measured at M0 and M12 for patients who had achieved a complete one-year free of lymphoma (*n* = 104 patients). As depicted in Fig. 3, the physical and mental aspects of HRQoL improved during this period. All components were significantly improved between M0 and M12 excepted for general and mental health. Although the HRQoL improved in general during the one year follow-up, some patients remained in a poor condition with 20 % of patients still displaying an MCS or PCS ≤ 50 at M12 (Fig. 3).

#### Professional and social changes

The majority of patients were in employment before treatment (61/115). However, 57 (93 %) went on sick leave during the active treatment phase and 45 of these returned to work (78.9 %) either in a full time (*n* = 32) or part-time capacity (*n* = 13). Among the total sample almost 10 % showed a reduction in financial resources and 4.3 % changed their home address. A change in marital status was infrequent over this period (1.7 %).

### Impact of psychological disorders on HRQoL and risk factors

A Pearson correlation matrix was constructed for each variable (PTSD, HAD-Depression, HAD-Anxiety, SF36-MCS, and SF36-PCS scores), measured at M0, M3, M6, M9 and M12 (Table 3). This matrix shows a constant connection between all of these variables. Bivariate analysis revealed that several factors were associated with the probability of developing at least one psychological disorder during one year of follow-up. These included gender (female), age (<60 years), histology (HL) and, more importantly, lower mental and physical HRQoL at M0. Thus, multivariate analysis showed that patients potentially at risk for developing at least one psychological disorder are females, patients diagnosed with HL, and patients with lower self-perceived mental HRQoL at M0 (Table 4).

**Table 3:** Pearson correlation coefficients for the scores of the PTSD checklist (at months 1, 6 and, 12), the hospital anxiety and depression scale (Months 3, 6, 9 and 12), and the SF-36 health survey (Months 1 and 12)

PTSD (M0)	PTSD (M6)	PTSD (M12)	HADA (M3)	HADA (M6)	HADA (M9)	HADA (M12)	HADD (M3)	HADD (M6)	HADD (M9)	HADD (M12)	SF36 MCS (M0)	SF36-PCS (M0)	SF36-MCS (M12)	SF36-PCS (M12)	
1	0.79649	0.73146	0.56455	0.55338	0.53787	0.59773	0.44034	0.39448	0.38274	0.47341	−0.51828	−0.41293	−0.59287	−0.54638	PTSD
	<.0001	<.0001	<.0001	<.0001	<.0001	<.0001	<.0001	<.0001	<.0001	<.0001	<.0001	<.0001	<.0001	<.0001	(M0)
	1	0.79187	0.58923	0.6597	0.65753	0.7029	0.41921	0.47819	0.48008	0.49995	−0.5599	−0.4472	−0.64859	−0.56404	PTSD
		<.0001	<.0001	<.0001	<.0001	<.0001	<.0001	<.0001	<.0001	<.0001	<.0001	<.00016	<.0001	<.0001	(M6)
		1	0.45231	0.52977	0.59971	0.64537	0.33995	0.39253	0.46842	0.54995	−0.38771	−0.30926	−0.69486	−0.54733	PTSD
			<.0001	<.0001	<.0001	<.0001	0.0004	<.0001	<.0001	<.0001	<.0001	0.0013	<.0001	<.0001	(M12)
			1	0.65131	0.70539	0.62431	0.42708	0.30713	0.29832	0.34666	−0.3969	−0.36314	−0.45017	−0.37952	HADA
				<.0001	<.0001	<.0001	<.0001	0.0009	0.0015	0.0002	<.0001	<.0001	<.0001	<.0001	(M3)
				1	0.74176	0.71154	0.40855	0.48015	0.47148	0.43145	−0.38351	−0.4053	−0.43999	−0.40748	HADA
					<.0001	<.0001	<.0001	<.0001	<.0001	<.0001	<.0001	<.0001	<.0001	<.0001	(M6)
					1	0.77938	0.47043	0.44302	0.55754	0.49862	−0.37517	−0.36301	−0.49752	−0.43972	HADA
						<.0001	<.0001	<.0001	<.0001	<.0001	<.0001	<.0001	<.0001	<.0001	(M9)
						1	0.38733	0.49511	0.51986	0.55888	−0.48623	−0.43266	−0.51551	−0.46204	HADA
							<.0001	<.0001	<.0001	<.0001	<.0001	<.0001	<.0001	<.0001	(M12)
							1	0.64098	0.58671	0.56799	−0.48358	−0.39613	−0.42135	−0.39067	HADD
								<.0001	<.0001	<.0001	<.0001	<.0001	<.0001	<.0001	(M3)
								1	0.57086	0.58342	−0.40282	−0.36483	−0.41359	−0.39385	HADD
									<.0001	<.0001	<.0001	<.0001	<.0001	<.0001	(M6)
									1	0.73002	−0.31758	−0.31682	−0.5145	−0.45989	HADD
										<.0001	0.0007	0.0007	<.0001	<.0001	(M9)
										1	−0.39559	−0.37602	−0.6048	−0.49074	HADD
											<.0001	<.0001	<.0001	<.0001	(M12)
											1	0.78154	0.49547	0.47986	SF36 MCS
												<.0001	<.0001	<.0001	(M0)
												1	0.46939	0.64959	SF36-PCS
													<.0001	<.0001	(M0)
													1	0.80799	SF36-MCS
														<.0001	(M12)
														1	SF36-PCS (M12)

**Table 4:** Bi-and multivariate analysis for the identification of groups at risk for developing at least one psychological disorder during one year of follow-up (*n* = 104)

	Crude OR	95 % CI	p - Value	Adjusted OR	95 % CI	*p* - Value
Gender						
Men	1.00 (Ref.)			1.00 (Ref.)		
Women	2.90	(1.35; 6.23)	0.0064	7.14	(1.95; 26.16)	0.003
Age						
≤ 60	1.00 (Ref.)			1.00 (Ref.)		
> 60	0.32	(0.14; 0.71)	0.0051	0.79	(0.11; 5.58)	0.8155
Level of education						
Lower educational status (≤high school degree)	1.00 (Ref.)			1.00 (Ref.)		
Higher educational status (>high school degree)	1.85	(0.88; 3.92)	0.1066	1.66	(0.52; 5.32)	0.3929
Occupational status						
In activity (employed)	1.00 (Ref.)			1.00 (Ref.)		
Without activity (retired, unemployed)	0.59	(0.28; 1.24)	0.1627	2.49	(0.45; 13.98)	0.2987
Histology						
Diffuse large B-cell lymphoma	1.00 (Ref.)			1.00 (Ref.)		
Hodgkin lymphoma	7.7	(2.25; 26.36)	0.0011	25.46	(4.00; 162.13)	0.0006
Other non-Hodgkin lymphoma	1.83	(0.77; 4.36)	0.1698	3.73	(0.87; 16.05)	0.0709
Charlson Comorbidity Index						
0	1.00 (Ref.)			1.00 (Ref.)		
1	0.33	(0.06; 1.67)	0.1786	0.69	(0.08; 6.03)	0.7396
≥ 2	0.57	(0.20; 1.66)	0.306	0.63	(0.14; 2.91)	0.5536
First-line treatment						
Intensified	1.00 (Ref.)			1.00 (Ref.)		
Conventional	0.73	(0.293; 1.82)	0.5014	0.39	(0.07; 2.07)	0.2682
Health-related quality of life (SF-36) at M0						
Physical component score	0.95	(0.93; 0.97)	<.0001	0.98	(0.94; 1.02)	0.375
Mental component score	1.07	(1.04; 1.09.)	<.0001	0.93	(0.89; 0.97)	0.0009

NOTE: Covariates were chosen with a cut-off value <0.20 in the bivariate analysis, except for the covariate first-line treatment

*Abbreviations*: *OR* Odds Ratio; *CI* Confidence Interval; *SF-36* 36-item short-form health survey

Model: adjusted for: gender, age, level of education, occupation, histology, Charlson comorbidity index, type of first-line treatment, health-related quality of life (mental and physical component score)

## Discussion

The aim of this prospective cohort study was to investigate the feasibility of using the AMA-AC procedure to monitor lymphoma survivors for any physical, psychological and social events that occurred during their first year after therapy. The implementation of the AMA-AC procedure showed not only that it could be feasibly used for this purpose but also that it is transferable into clinical daily practice.

All patients voluntarily entered into the study and accepted the conditions of the program, that they would be mainly monitored by their GP and the NC, with the oncologist being available only upon request. This unrestricted approval could be due to the climate of confidence established between the patient and the NC during the active phase of treatment as part of the AMA process [20]. Indeed, we believe that AMA during the active phase (now designated as AMA1 in our institution) played an important role in the success of this AMA-AC program, and that AMA1 and AMA-AC are highly complementary (all patients enrolled in AMA-AC were initially enrolled in AMA1). The fact that the majority of our patients were well-educated and young (a median age of 55 years) may have also facilitated not only acceptance but also adherence. This selection bias could raise some concerns with respect to the generalizability of our findings. Thus, it remains possible that in a wider population a loss of adherence could occur concerning one or several components of the procedure such as attending GP appointments, taking NC calls or returning self-reported questionnaires. It is also important to note that all GPs participated in the AMA-AC, a total of 113 GPs (2 of whom each monitored 2 patients). This high rate of GP acceptance (113/113) probably reflects the motivation of GPs to contribute to survivorship management in association with the oncology hospital unit according to the “shared care” model. AMA-AC is a time-consuming procedure, requiring a mean of 55 min per quarter per patient, without taking into account the time spent by the patient in completing the auto-questionnaires (PCL and SF-36). Of these 55 min, the largest time contribution was from the NC (30 min) followed by the GP (15 min) and finally the oncologist (10 min). Compared to the standard surveillance performed in our department (a 30 min visit every 3 months for the first year, then every 6 months for 5 years), there was a significant reduction in medical time with the oncologist (30 % reduction). The fact that the total time spent for patient management in AMA-AC was higher that than of our standard surveillance procedure would be expected to correlate with the superiority of AMA-AC in gathering information of different types and from different sources. Medico-economic evaluation of AMA-AC is beyond the scope of our study. However, one can speculate that the increased total time spent for patient management might be largely counterbalanced by the decrease in transportation costs. It is also possible that the limitation of visits would result in decreased absenteeism and subsequently an improved productivity for young and professionally active patients. These questions deserve more specific investigation.

This study has shown that AMA-AC could be an effective procedure for detecting physical events during the early trajectory of lymphoma survivorship. Until now, the occurrence or persistence of morbid manifestation had not been thoroughly examined during this period by prospective studies. Our prospective study shows a high occurrence of disabling symptoms, with those related to the treatment of arthralgia as the most frequent (64 %). We also found an unexpectedly high rate of ulcer and gastritis symptoms (17 %), probably due to corticosteroids administered during the active treatment phase. The high rate of infection (about 40 %) occurred in the context of moderate or mild hypogammaglobulinemia, suggesting the presence of other mechanisms of immunosuppression, perhaps due to profound and durable B-cell depletion induced by rituximab. Sexual dysfunction was also frequent, as previously reported [38]. However, the most disabling complication was neuropathy (24 %), as also previously reported [39, 40].

Newly-diagnosed comorbidities were unexpectedly high, particularly cardiovascular diseases and second cancers (almost 20 % of patients). This unprecedented finding is intriguing since the majority of our patients were relatively young, in good health, with a low CCI. It is possible that comorbidities have been underestimated in the early trajectory of lymphoma patients treated with R-CHOP or R-CHOP-derived. The CT scan performed at 6 months allowed the detection of pancreatic tumours (*n* = 2), asymptomatic severe biliary disease (*n* = 1) and a pulmonary embolism (*n* = 1). However, in terms of quality of lymphoma surveillance the CT scan was poor, in agreement with other studies which do not support the use of routine CT scan imaging for the follow-up of DLBCL [15]. In the context of the frequent non-haematological complications observed, AMA-AC was found to be helpful in facilitating the coordination between the GP, the oncologist and the relevant specialist in order to define priorities and new trajectories. However, it is interesting to note that, in most cases, even when these complications were diagnosed by the GP, the oncologist was requested before the patient was referred to another specialist. The reason for this is that GPs tend to relate symptoms to relapse before considering the possibility of associated diseases, including the most frequent (vascular complications and second cancers). This experience places the AMA unit in a central position for general medical management, this role being reinforced by the special links established with the patient along their trajectory.

Furthermore, based on psychometric measurements it seems that AMA-AC may also be an effective procedure for detecting psychological disorders (e.g., anxiety, depression and PTSD). These complications are thought to be underestimated in cancer survivorship, mainly because hospital-based follow-up is poorly adapted. This is due in part to heavy overbooking of the oncology unit but is also related to the lack of education or even interest of oncologists in onco-psychology. This latter statement is reflected in the negative perception of patients in terms of the role of the oncologist in managing such complications [41–43], and suggests the need for the development of new interventions such as AMA-AC to address the psychosocial and physical concerns throughout the course of the cancer trajectory [41]. Our study found that 42 % of patients presented with at least one psychological disorder during the first year of follow-up. These results are in agreement with retrospective transversal and longitudinal studies dealing with both HL and NHL [2, 3, 44–46]. However, our prospective study shows that the development of psychological disorders in lymphoma survivorship changes over time and that psychological support is essentially needed at the beginning of the after-cancer trajectory as has been described for other cancers including breast and ovarian cancers [47].

AMA-AC also appears to be an effective method for monitoring social changes including absenteeism and return to work. Among the patients in employment, 73 % returned to work within 12 months. This percentage was higher in HL compared to NHL, as previously reported [48], and this rate is also in agreement with a recent Danish registry study (1,741 patients) [4]. Due to the relatively low number of patients monitored in our study, it is not possible to draw any conclusions about the possible role of AMA-AC in facilitating return to work. However, we found AMA-AC to help facilitate communication and coordination between oncologists, NCs, GPs and occupational health professionals, which is helpful for adapting the return to work to the patient’s physical and mental capacities.

AMA-AC appears to be a simple procedure for monitoring HRQoL in routine practice. In lymphoma patients, HRQoL has been mainly investigated through cross-sectional studies and less frequently in prospective studies. From these previous studies, it appears that up to one third of patients experience an alteration in their HRQoL which is severe compared to other cancers, some of which are more aggressive [49], and is often enduring [1]. The present study shows that, although HRQoL improved between M0 and M12, it remained significantly affected one year after completion of treatment in about 20 % of patients even when the PCS and MCS scores were not adjusted for age.

Multivariate analysis allowed us to identify a number of risk factors associated with the occurrence of psychological disorders. HL appears to be a significant parameter, as has previously been suggested [50]. Female gender also appears to be an independent risk factor, as suggested by a recent meta-analysis [51]. Lower MCS after completion of chemotherapy is also highly predictive of the occurrence of psychological disorders. These results suggest that patients with a combination of risk factors (female, advanced HL, with lower MCS after therapy - in most cases associated with poor tolerability) would benefit from adequate psychological support during the early trajectory.

Our study suffers from several limits. First, our cohort presented some degree of heterogeneity in terms of histology subtype, chemotherapy and risk of relapse, even though all patients presented with advanced diseases and were treated with prolonged anthracycline-based chemotherapy. Moreover, several characteristics reflect a patient selection bias such as age, education level and fitness. These parameters, as well as the introduction of AMA1 in our institution, may have affected the acceptance and adherence to the AMA-AC procedure. The next step in determining the usability of AMA-AC is to assess the medico-economics and to conduct a satisfaction survey for patients and GPs. We are currently performing several studies to investigate these important aspects.

## Conclusion

AMA-AC appears to be a promising alternative to the standard follow-up for lymphoma survivorship surveillance. It is a feasible and reproducible procedure, which was found to be very effective in detecting physical events (including new non-haematological diseases), psychological disorders and social problems (including return to work). AMA-AC represents a “shared care” model which attributes the premium roles to the NC and GPs. Further studies comparing AMA-AC with the oncologist-based follow-up procedure are now needed to establish AMA-AC as a standard surveillance method for both non-haematological and haematological malignancies.

## Additional file

Additional file 1: **List of medical complications administered by the general practitioner (GP).** (DOCX 32 kb)

## Abbreviations

AMA-AC: Ambulatory medical assistance - after cancer
ABVD: Doxorubicin (adriamycin), bleomycin, vinblastine, dacarbazine
BEACOPP: Bleomycin, etoposide, doxorubicin (adriamycin), cyclophosphamide, vincristine, procarbazine, prednisone
CCI: Charlson comorbidity index
CHOP: Cyclophosphamide, adriamycin, vincristine, prednisone
CHVP: Cyclophosphamide, adriamycin, etoposide, prednisone
CRF: Clinical report form
DLBCL: Diffuse large B-cell lymphoma
GP: General practitioner
HAD-A: Hospital anxiety and depression - anxiety
HAD-D: Hospital anxiety and depression – depression
HADS: Hospital anxiety and depression scale
HL: Hodgkin lymphoma
HRQoL: Health-related quality of life
MCS: Mental component score
NC: Nurse coordinator
NHL: Non-Hodgkin lymphoma
PCL: Post-traumatic stress disorder checklist
PCS: Physical component score
PTSD: Post-traumatic stress disorder
R-ACVBP: Rituximab, doxorubicin, cyclophosphamide, vindesine, bleomycin, and prednisone
R-CHOP: Rituximab, cyclophosphamide, adriamycin, oncovin®, prednisone
R-COPADM: Rituximab, cyclophosphamide, oncovin®, prednisone, doxorubicin, methotrexate high doses
