# Supplementary material for: Ambulatory Medical Assistance - After Cancer (AMA-AC): A model for an early trajectory survivorship survey of lymphoma patients treated with anthracycline-based chemotherapy
Source: BMC Cancer. 2015 Oct 24;15:781. doi: 10.1186/s12885-015-1815-7 (PMC4619467; doi:10.1186/s12885-015-1815-7)
Supplement: Additional file 1: — List of medical complications administered by the general practitioner (GP). (DOCX 32 kb) [file 12885_2015_1815_MOESM1_ESM.docx]

**Additional file 1: List of medical complications administered by the general practitioner (GP)**

| **Medical Complications** | **Yes** | **No** | **Comments** | **Medical Complications** | **Yes** | **No** | **Comments** |
| --- | --- | --- | --- | --- | --- | --- | --- |
| Suspicion of Relapse |  |  |  | Gastro-Intestinal (except Tumours) |  |  |  |
| General Signs and Symptoms |  |  |  | Mucositis |  |  |  |
| Fever |  |  |  | Gastritis or Ulcer |  |  |  |
| Sweating |  |  |  | Others |  |  |  |
| Weight Loss |  |  |  | Prolapse: Rectal |  |  |  |
| Eye and Adnexa |  |  |  | Incontinence (Faecal) |  |  |  |
| Visual Acuity |  |  |  | Genito-Urinary Tract |  |  |  |
| Musculoskeletal and Connective Tissue |  |  |  | Prostate Tumour |  |  |  |
| Arthralgia |  |  |  | Other Tumours |  |  |  |
| Osteoporosis |  |  |  | Prolapse: Genital |  |  |  |
| Others |  |  |  | Incontinence (Urine) |  |  |  |
| Cardiovascular |  |  |  | Others |  |  |  |
| Myocardial Infarction (MI) |  |  |  | Sexual |  |  |  |
| Coronary Heart Diseases (other than MI) |  |  |  | Contraception |  |  |  |
| Congestive Heart Failure |  |  |  | Pregnancy |  |  |  |
| Cardiac Rhythm Disorders |  |  |  | Menopause |  |  |  |
| Venous Thrombosis |  |  |  | Libido Decrease |  |  |  |
| Arterial Thrombosis |  |  |  | Erectile Dysfunction |  |  |  |
| Others (e.g., Pericarditis) |  |  |  | Others |  |  |  |
| Skin and Subcutaneous Tissue |  |  |  | Infections |  |  |  |
| Endocrine System |  |  |  | Pulmonary Infections |  |  |  |
| Thyroid |  |  |  | ENT Infections |  |  |  |
| Diabetes Mellitus |  |  |  | Urinary Tract Infections |  |  |  |
| Others |  |  |  | Others |  |  |  |
| Abbreviations: ENT; ears, nose, and throat; MI; myocardial infarction. | | | | | | | |
